# Association of activated Gα_q_ to the tumor suppressor Fhit is enhanced by phospholipase Cβ

**DOI:** 10.1186/s12885-015-1802-z

**Published:** 2015-10-24

**Authors:** Hao Zuo, Yung H. Wong

**Affiliations:** Division of Life Sciences, and the Biotechnology Research Institute, Hong Kong University of Science and Technology, Clear Water Bay, Kowloon, Hong Kong China; State Key Laboratory of Molecular Neuroscience, and the Molecular Neuroscience Center, Hong Kong University of Science and Technology, Clear Water Bay, Kowloon, Hong Kong; Present address: Department of Pharmacology, University of Texas Southwestern Medical Center, 5323 Harry Hines Blvd, Dallas, TX 75390 USA

**Keywords:** Fhit, G protein, Phospholipase Cβ, Tumor suppression

## Abstract

**Background:**

G proteins are known to modulate various growth signals and are implicated in the regulation of tumorigenesis. The tumor suppressor Fhit is a newly identified interaction partner of G_q_ proteins that typically stimulate the phospholipase C pathway. Activated Gα_q_ subunits have been shown to interact directly with Fhit, up-regulate Fhit expression and enhance its suppressive effect on cell growth and migration. Other signaling molecules may be involved in modulating Gα_q_/Fhit interaction.

**Methods:**

To test the relationship of PLCβ with the interaction between Gα_q_ and Fhit, co-immunoprecipication assay was performed on HEK293 cells co-transfected with different combinations of Flag-Fhit, Gα_16_, Gα_16_QL, pcDNA3 vector, and PLCβ isoforms. Possible associations of Fhit with other effectors of Gα_q_ were also demonstrated by co-immunoprecipitation. The regions of Gα_q_ for Fhit interaction and PLCβ stimulation were further evaluated by inositol phosphates accumulation assay using a series of Gα_16/z_ chimeras with discrete regions of Gα_16_ replaced by those of Gα_z_.

**Results:**

PLCβ1, 2 and 3 interacted with Fhit regardless of the expression of Gα_q_. Expression of PLCβ increased the affinities of Fhit for both wild-type and activated Gα_q_. Swapping of the Fhit-interacting α2-β4 region of Gα_q_ with Gα_i_ eliminated the association of Gα_q_ with Fhit without affecting the ability of the mutant to stimulate PLCβ. Other effectors of Gα_q_ including RGS2 and p63RhoGEF were unable to interact with Fhit.

**Conclusions:**

PLCβ may participate in the regulation of Fhit by G_q_ in a unique way. PLCβ interacts with Fhit and increases the interaction between Gα_q_ and Fhit. The Gα_q_/PLCβ/Fhit complex formation points to a novel signaling pathway that may negatively regulate tumor cell growth.

**Electronic supplementary material:**

The online version of this article (doi:10.1186/s12885-015-1802-z) contains supplementary material, which is available to authorized users.

## Background

The fragile *FHIT* gene at the chromosomal fragile site FRA3B is often regarded as an early target of DNA damage in precancerous cells. Its gene product, the ubiquitously expressed Fhit (Fragile Histidine Triad) protein, is a member of the HIT (histidine triad) superfamily with three signature histidines in the conserved nucleoside binding motif. Fhit binds and hydrolyzes various dinucleoside polyphosphates (such as Ap_3_A, Ap_4_A, Ap_3_G and Cp_3_G) into two nucleotides where one is a nucleoside monophosphate [1]. The preferred substrate of Fhit is Ap_3_A (diadenosine 5′,5‴-P1,P3-triphosphate) which is hydrolyzed to AMP and ADP. Interestingly, Fhit acts as a tumor suppressor and its down-regulation is associated with different tumors including lung cancers [2]. Re-expression of Fhit in Fhit-deficient tumor cells can notably suppress tumor development [3–5]. Several theories of tumor suppression have been proposed for Fhit with the overarching idea of Fhit acting as a genome “caretaker” [6, 7]. Reduced Fhit expression has indeed been shown to increase DNA replication stress and genome alterations as a result of a decreased intracellular thymidine triphosphate (dTTP) level [8]. Moreover, Fhit is apparently involved in suppressing lung tumor cell migration/invasion by down-regulating the expression of matrix metalloproteinase 2/9 [9]. Despite considerable efforts, the precise mechanism by which Fhit exerts its tumor suppressive function remains elusive. The dinucleoside polyphosphate hydrolase activity of Fhit seemingly plays a trivial role in tumor suppression [10].

A number of studies have revealed unsuspecting binding partners of Fhit that may provide linkages to processes that contribute to tumor eradication such as cellular oxidation and apoptosis. Fhit-interacting molecules include β-catenin [11], ferredoxin reductase [12], Src tyrosine kinase [13] and ubiquitin conjugating enzyme 9 [14]. More recently, we have demonstrated that Fhit can distinguish between inactive and active signal transducing Gα subunits of the G_q_ family [15]. This finding is intriguing as it may link Fhit to Gα_q_-dependent signals that modulate a variety of cellular events. Fhit-mediated suppression of epithelial-mesenchymal transition in bronchial cells involves the epidermal growth factor receptor (EGFR), Src, and extracellular signal-regulated kinase (ERK) [16] that have all been shown to be activated or transactivated by Gα_q_ [17, 18]. In human colon cancer cell lines, Fhit inhibits cell proliferation by attenuating the nuclear factor κB (NFκB) pathway [19] which can be stimulated by G_q_-coupled receptors [20]. It is also noteworthy that sustained activation of the G_q_ pathway often leads to mitogenesis in a variety of cell types [21] with disparate mechanisms of regulating cell cycle progression [22]. The opposing roles of Fhit and Gα_q_ tend to suggest that they may exert counteracting actions on each other. However, Fhit neither inhibits nor enhances Gα_q_-induced signals [15] whereas its own expression becomes translationally up-regulated by activated Gα_q_ [23]. Given the links between G_q_ signaling and mitogenesis as well as those between Fhit and tumor suppression are well-established, it seems reasonable to expect that the binding of Fhit to activated Gα_q_ would impart functional consequences. Since the canonical signaling pathway of all Gα_q_ subfamily members (Gα_q_, Gα_11_, Gα_14_ and Gα_15/16_) is the activation of phospholipase Cβ (PLCβ), we further explored the influence of PLCβ on the formation of Fhit/activated Gα_q_ complexes. Here, we report that different isoforms of PLCβ can also associate with Fhit in the absence of Gα_q_ activation.

## Methods

### Reagents

The human cDNAs of various Gα subunits were obtained from Guthrie Research Institute (Sayre, PA). Wild-type Fhit in pCMV-SPORT6 was purchased from Invitrogen (Carlsbad, CA). Gα_16/z_ chimeras were constructed by overlapping PCR which swapped the corresponding regions of Gα_16_ with Gα_z_ as described previously [15]. Cell culture and Lipofectamine PLUS reagents, and anti-Fhit antibody were purchased from Invitrogen (Carlsbad, CA). Anti-Gα_16_ was obtained from Gramsch Laboratories (Schwabhausen, Germany). Anti-Gα_q/11_ antibody was purchased from Calbiochem (San Diego, CA). Anti-α-tubulin, anti-HA, and anti-Flag antibodies as well as anti-HA affinity gel were from Sigma-Aldrich (St. Louis, MO). Antisera against PLCβ1/2/3 were purchased from Santa Cruz Biotechnology (Santa Cruz, CA). Other antibodies were purchased from Cell Signaling Technology (Danvers, MA). Protein G-agarose was from Thermo Fisher Scientific (Rockford, IL). ECL kit was from Amersham Biosciences (Piscataway, NJ).

### Cell culture and Co-immunoprecipitation

HEK293 cells were obtained from the American Type Culture Collection (CRL-1573, Rockville, MD). They were maintained in Eagle’s minimum essential medium at 5 % CO_2_, 37 °C with 10 % fetal bovine serum, 50 units/mL penicillin and 50 μg/mL streptomycin. Transfection was performed according to the manual of Lipofectamine^®^ transfection reagent. One day later, cells were lysed in ice-cold RIPA buffer (25 mM HEPES at pH 7.4, 0.1 % SDS, 1 % Nonidet P-40, 0.5 % sodium deoxycholate, 1 mM dithiothreitol, 200 μM Na_3_VO_4_, 4 μg/mL aprotinin, 100 μM phenylmethylsulfonyl fluoride, and 2 μg/mL leupeptin). Cell lysates were incubated with a primary antiserum with rotation at 4 °C overnight, and then incubated in 30 μL protein G-agarose (50 % slurry) at 4 °C for 4 h. Alternatively, the cell lysates were incubated in 30 μL anti-Flag affinity agarose gel (50 % slurry) at 4 °C for 4 h. Immunoprecipitates were washed with ice-cold RIPA buffer (400 μL) for four times, resuspended in 50 μl RIPA buffer and 10 μl 6× sample buffer and then boiled for 5 min. Target proteins in the immunoprecipitates were analyzed by Western blots. Signal intensities of the immunoreactive bands were quantified using Image J software, version 1.38× (National Institutes of Health, USA).

### Western blotting analysis

Protein samples were resolved on 12 % SDS-polyacrylamide gels and transferred to Osmonics nitrocellulose membrane. Resolved proteins were detected by their specific primary antibodies and horseradish peroxidase-conjugated secondary antisera. The immunoblots were visualized by chemiluminescence with the ECL kit from Amersham, and the images detected in X-ray films were quantified by densitometric scanning using the Eagle Eye II still video system (Stratagene, La Jolla, CA, USA).

### Inositol phosphates accumulation assay

HEK293 cells were seeded at a density of 2×10^5^ cells/well into 12-well plates. Various cDNAs at a concentration of 0.5 μg/well were transiently transfected into the cells using Lipofectamine^®^ transfection reagents. One day after transfection, cells were labeled with inositol-free Dubecco’s modified Eagle’s medium (DMEM; 750 μL) containing 5 % FBS and 2.5 μCi/mL *myo*-[^3^H]inositol overnight. The labeled cells were then washed once with IP_3_ assay medium (20 mM HEPES, 5 mM LiCl, serum-free DMEM) and then incubated with 500 μl IP_3_ assay medium at 37 °C for 1 h. Reactions were stopped by replacing the assay medium with 750 μL ice-cold 20 mM formic acid and the lysates were kept in 4 °C for 30 min before the separation of [^3^H]inositol phosphates from other labeled species by sequential ion-exchange chromatography as described previously [24].

### Statistical analysis

Data were expressed as the mean ± S.E. of at least three independent sets of experiments. The probability of an observed difference being a coincidence was evaluated by Dunnett *t* test. Differences at values of *P* < 0.05 were considered significant (* *P* < 0.05).

## Results

**Fig. 1:**
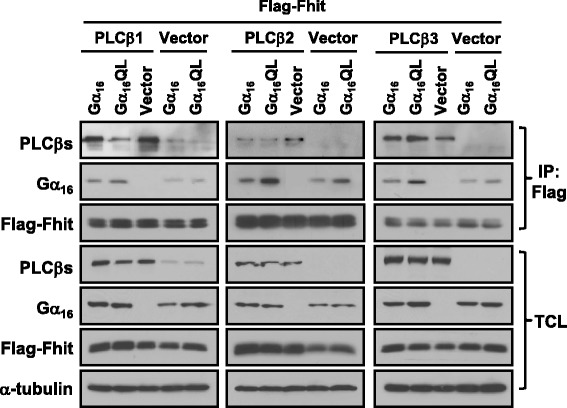
PLCβs enhance the interaction between Fhit and Gα_q_. HEK293 cells were co-transfected with different combinations of Flag-Fhit, Gα_16_, Gα_16_QL, pcDNA3 vector, PLCβ1, PLCβ2 or PLCβ3. One day after transfection, cell lysates were prepared and immunoprecipitated with anti-Flag affinity gel. PLCβ1, 2, 3, Gα_16_, Fhit and α-tubulin of the co-immunoprecipitation (IP) assay and total cell lysate (TCL) were determined by Western blotting

**Fig. 2:**
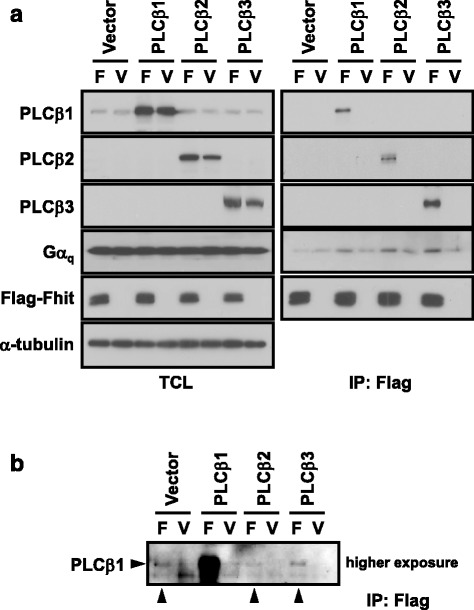
Fhit interacts with PLCβs. **a** HEK293 cells were transfected with pcDNA3 vector, PLCβ1, PLCβ2 or PLCβ3 in combination with Flag-Fhit (F) or pFlag-CMV2 (V) vector. Following expression for 1 day, cells were lysed and subjected to co-immunoprecipitation assay with anti-Flag affinity gel. The levels of Fhit and PLCβs were examined by Western blotting. **b** In the co-immunoprecipitation assay in a, a longer exposure of the anti-PLCβ1 blot showed that endogenous PLCβ1 (indicated by a horizontal arrow) was pulled down by Fhit (indicated by vertical arrows)

We have previously shown that Fhit directly interacts with activated members of the Gα_q_ family (Gα_q_, Gα_14_, and Gα_16_) via their α2-β4 region without affecting Gα_q_-induced PLCβ activation [15]. As PLCβ also interacts with the α2 region of the activated Gα_q_ [25], we asked whether PLCβ can compete with Fhit for the activated Gα_q_ in co-immunoprecipitation assays. HEK293 cells were co-transfected with Flag-tagged Fhit and wild-type or activated Gα_16_ (Gα_16_QL) with or without PLCβ1, PLCβ2 or PLCβ3. Because activated Gα_q_ signaling always increase the expression levels of Fhit [23], we adjusted the Fhit cDNA amount for transfection to obtain similar Fhit expression levels. In order to facilitate the assessment of expression and to minimize interference by endogenous Gα_q_ subunits, we have opted for using Gα_16_ as a representative Gα_q_ member. In vector transfected cells, Fhit pulled down detectably more Gα_16_QL than wild-type Gα_16_ (Fig. 1), as reported previously [15]. Overexpression of PLCβ1, PLCβ2 or PLCβ3 increased the affinities of both wild-type and activated Gα_16_ for Fhit (*cf* lanes 1, 2 and lanes 4, 5 in the three panels of the second row of Fig. 1). Thus PLCβs did not appear to compete with Fhit for activated Gα_16_. Instead, the presence of PLCβs apparently enhanced or stabilized the association of Fhit and Gα_16_. More interestingly, all three isoforms of PLCβ were also detected in the Fhit-immunoprecipitates (Fig. 1). To test if Fhit can form complexes with PLCβs, HEK293 cells were transfected with vector or PLCβ1-3 in combination with Flag-Fhit or Flag vector. All three isoforms of PLCβ were able to co-immunoprecipitate with Fhit (Fig. 2a). Since HEK293 cells endogenously express PLCβ1 (Fig. 2a, upper left panel), Flag-Fhit might be able to pull down endogenous PLCβ1. Longer exposure of the anti-PLCβ1 blot indeed revealed the presence of endogenous PLCβ1 in the immunoprecipitates of Fhit (Fig. 2b). Moreover, as compared to the Flag vector control, Fhit could pull down endogenous wild-type Gα_q_ when one of the PLCβ isoforms was overexpressed (*cf* lane 1 and lanes 3, 5, and 7 of the fourth row on the right in Fig. 2a). These findings indicate that PLCβs may interact with Fhit and increase the association between Fhit and Gα_q_. We have previously reported that increased cell proliferation by Gα_q_ activation is suppressed in the presence of Fhit [15]. Not surprisingly, we also found that PLCβ3 overexpression alone was sufficient to trigger a higher cell growth rate and Fhit co-expression significantly decreased PLCβ3-induced cell proliferation (data not shown). Therefore, Fhit appears to be capable of suppressing G_q_-PLCβ mediated cell proliferation.

**Fig. 3:**
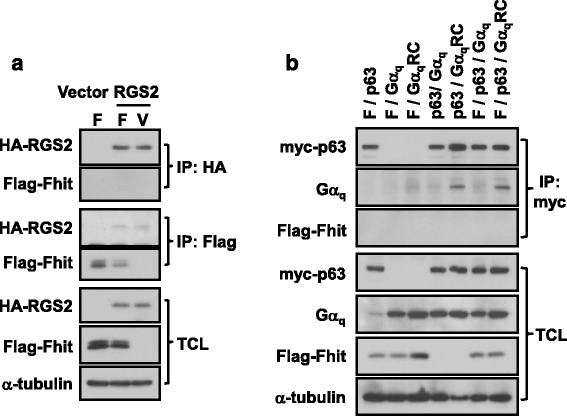
Fhit does not interact with RGS2 or p63RhoGEF. **a** HEK293 cells were transfected with pcDNA3 vector or HA-tagged RGS2 in combination with pFlag-CMV2 (V) or Flag-Fhit (F). Cell lysates were subjected to co-immunoprecipitation assay with anti-Flag or anti-HA affinity gel. RGS2, Gα_q_, Fhit and α-tubulin were detected by Western blotting. **b** HEK293 cells were transfected with different combinations of Flag-Fhit, myc-p63RhoGEF and Gα_q_ or Gα_q_RC. After 1 day, cells were subjected to the co-immunoprecipation with anti-myc affinity gel. The immunoprecipitates and total cell lysates were analyzed by Western blot

If PLCβ binds to Fhit via activated Gα_q_, other proteins known to associate with Gα_q_ may also co-immunoprecipitate with Fhit. Besides PLCβs, activated Gα_q_ also interacts with other proteins such as RGS2 [26] and p63RhoGEF [27]. Unlike PLCβs, Fhit did not interact with HA-tagged RGS2 in the co-immunoprecipitation assay using anti-Flag or anti-HA affinity gel (Fig. 3a). Similarly, in transfected HEK293 cells expressing various combinations of Flag-Fhit, myc-tagged p63RhoGEF, Gα_q_ and constitutively active Gα_q_RC, myc-p63RhoGEF pulled down Gα_q_RC but not Fhit (Fig. 3b). Moreover, the expression of Fhit did not affect the interaction between Gα_q_RC and p63RhoGEF (Fig. 3b). These results suggest that the ability of PLCβs to associate with Fhit and increase the interaction between Fhit and Gα_q_ may be specific.

**Fig. 4:**
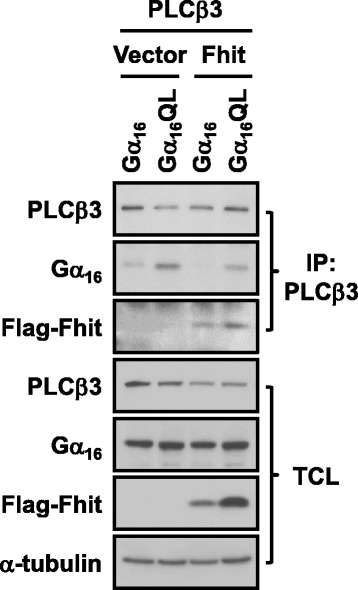
Fhit does not enhance the association of Gα_16_QL with PLCβ3. Gα_16_ or Gα_16_QL was co-transfected into HEK293 cells with pFlag-CMV2 (Vector) or Flag-Fhit in combination with PLCβ3. One day after transfection, cell lysates were immunoprecipitated with anti-PLCβ3 antibody and Protein G agrose, and subjected to Western blot analysis

Since overexpression of PLCβs appeared to enhance the interaction between Fhit and Gα_16_ (Fig. 1), we assessed whether Fhit can reciprocally enhance the interaction of PLCβ with Gα_q_ members. We performed co-immunoprecipitation assay with anti-PLCβ3 antiserum because it has the best specificity among the different anti-PLCβ antisera. Gα_16_QL as well as Fhit was co-immunoprecipitated with PLCβ3 (Fig. 4). Co-expression of Fhit appeared to weaken the interaction between Gα_16_QL and PLCβ3 (Fig. 4*cf* lanes 2 and 4 of row two). The reduction of Gα_16_QL in the PLCβ3-immunoprecipitates was not due to variations in the expression levels or pull down efficiency, as these parameters were essentially similar in the different samples (Fig. 4 rows one and five).

**Fig. 5:**
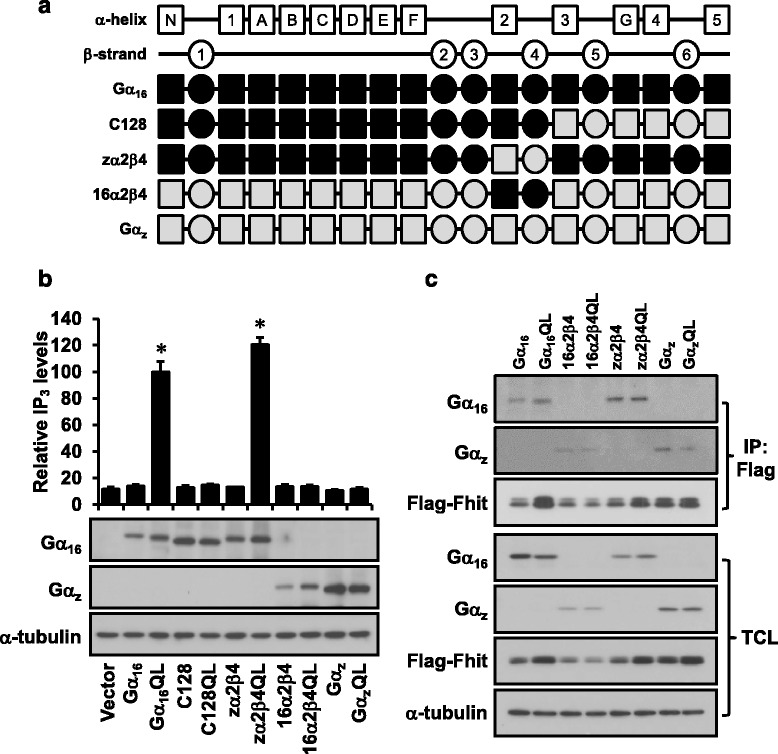
The chimera zα2β4 stimulates PLCβ but does not interact with Fhit. **a** Schematic representation of the zα2β4 and 16α2β4 chimeras. The linearized secondary structure of Gα_q_ (filled with white) includes a helical domain (helices A-G) and a GTPase domain (helices 1–5 and strands 1–6). In the secondary structures of Gα_16_, Gα_z_, C128, zα2β4 or 16α2β4, the sequences from Gα_16_ are filled with black and those from Gα_z_ are filled with gray. **b** Inositol phosphates accumulation assays were performed in COS-7 cells transfected with the wild-type or constitutively active mutants of Gα_16_, Gα_z_, C128, zα2β4 or 16α2β4. The relative IP_3_ production was quantified. The expressions of the chimeras were examined by the Western blot. * Gα_16_QL and zα2β4QL significantly increased the IP_3_ production (Dunnett’s *t* test, *P* < 0.05). **c** HEK293 cells were transiently co-transfected with Flag tagged Fhit and the wild-type or constitutively active mutants of Gα_16_, Gα_z_, zα2β4 or 16α2β4. Cell lysates were immunoprecipitated with anti-Flag agarose affinity gel (*upper panels*). Expression levels of Gα_16_, Gα_z_, Flag-Fhit and α-tubulin in the total cell lysate were detected by western blotting (*lower panels*)

By using a series of Gα_16/z_ chimeras with discrete regions of Gα_16_ replaced by those of Gα_z_ (a Gα_i_ subfamily member which does not interact with Fhit), we have previously identified the α2-β4 region of Gα_16_ as critical for Fhit interaction [15]. Interestingly, the α2 region of Gα_q_ is seemingly involved in binding to PLCβ [25]. To further investigate the associations among Gα_q_, PLCβ and Fhit, we constructed two new chimeras named zα2β4 (Gα_16_ backbone with α2β4 region from Gα_z_) and 16α2β4 (Gα_z_ backbone with α2β4 region from Gα_16_), wherein the α2-β4 region of Gα_16_ or Gα_z_ was swapped with each other (Fig. 5a). As shown in Fig. 5b, both wild-type and constitutively active mutants of the zα2β4 and 16α2β4 chimeras can be expressed in HEK293 cells to levels that were comparable to those of Gα_16_, Gα_z_, or C128 (a previously characterized chimera with the C-terminal 128 residues of Gα_16_ swapped with the corresponding sequences of Gα_z_). Because the Gα-specific antibodies are N-terminal targeting, the zα2β4 and 16α2β4 chimeras were recognized by anti-Gα_16_ and anti-Gα_z_ antisera, respectively. When examined for their ability to stimulate PLCβ, activated zα2β4QL efficiently stimulated the formation of inositol phosphates to an extent similar to that of Gα_16_QL (Fig. 5b). The constitutively active Gα_z_QL did not activate PLCβ because Gα_z_ belongs to the Gα_i_ subfamily (Fig. 5b). Since neither 16α2β4QL nor C128QL was able to stimulate PLCβ (Fig. 5b), it indicated that the α2-β4 region of Gα_16_ alone was not sufficient to stimulate PLCβ.

A hallmark of Fhit/Gα_q_ interaction is the enhanced association with the activated Gα_q_ subunits over their wild-type counterparts [15]. Activation state-dependent interaction with Fhit was reproducibly observed in the present study (Fig. 1 and Additional file 1: Figure S1) and this feature was therefore used as an indicator of Fhit association with the chimeras. In co-immunoprecipitation assays, both the wild-type and constitutively active mutant of zα2β4 were pulled down by Flag-Fhit to similar extents (Fig. 5c), despite the fact that zα2β4QL was fully capable of stimulating PLCβ (Fig. 5b). Thus the α2β4 region of activated Gα_16_ is required by Fhit association but not by PLCβ activation. In the control groups, more Gα_16_QL was detected in the Fhit-immunoprecipitates than wild-type Gα_16_ whereas both Gα_z_ and Gα_z_QL were hardly detected (Fig. 5c). These control groups produced the same results as in our previous report [15]. The presence of 16α2β4 or 16α2β4QL in the Fhit-immunoprecipitates was even weaker than those Gα_z_ (Fig. 5c), suggesting that this chimera could not be recognized by Fhit. It should be noted that activation state-dependent association with Fhit has previously been demonstrated for the C128 chimera [15], while C128QL was not able to stimulate PLCβ (Fig. 5b). According to the above results of zα2β4, 16α2β4 and C128, the capability of activated Gα_16_QL to associate with Fhit did not affect its ability to stimulate PLCβ and it may explain why PLCβ enhanced the association of Fhit and Gα_q_ instead of competing with Fhit for Gα_q_,

## Discussion

The diversity of pathways downstream of G_q_ has endowed mammalian cells with a complex signaling network for the delicate regulation of a multitude of biological effects, with some responses being cell type-specific. One example is the fact that activation of G_q_ leads to proliferation in some cells while it induces apoptosis in other cell types [22]. The tumor suppressor Fhit taps into the G_q_ signaling network through its ability to associate with activated Gα_q_ [15, 23]. Interestingly, Fhit suppresses G_q_-mediated cell growth in H1299 lung cancer cells via an unknown mechanism [15, 23]. Here, we showed that the canonical Gα_q_ effector PLCβ can form a complex with activated Gα_q_ and Fhit, and can increase the overall association of the latter two proteins. Many tripartite or even higher order complexes involving Gα_q_ subunits are known to exist [28]. For instance, Gα_q_ can simultaneously bind to p63RhoGEF and RhoA [27], to G protein-coupled receptor kinase 2 (GRK2) and Gβγ [29], as well as to ADP-ribosylation factor 6 (ARF6) and ARNO (a GEF for ARF6) [30]. Hence, the existence of a Gα_q_/PLCβ/Fhit complex seems plausible.

Given the prior demonstration that direct binding of Fhit to activated Gα_q_ does not affect PLCβ activity [15], the ability of PLCβ to form a complex with Fhit/Gα_q_ is rather surprising. All three PLCβ isoforms (PLCβ1, 2 and 3) tested as well as the endogenously expressed PLCβ1 could be detected in the immunoprecipitates of Fhit in the absence of Gα_q_ overexpression (Fig. 2). Therefore, it is possible that PLCβ can directly interact with Fhit. Although the PLCβ association with Fhit may also occur via binding to endogenous Gα_q_ subunits, the interaction between PLCβ and Fhit appears to be specific because other Gα_q_ effectors such as RGS2 and p63RhoGEF did not interact with Fhit even in the presence of activated Gα_q_ (Fig. 3). PLCβs have weak affinities for inactive Gα_q_ subunits (EC_50_ at ~10 μM range) [31] but this basal interaction between PLCβ3 and inactive Gα_q_ could be detected (Fig. 4). The increased affinity of Fhit with inactive Gα_q_ upon overexpression of PLCβs (Figs. 1 and 2b) may result from the basal interactions between PLCβs and inactive Gα_q_ and the interaction between PLCβ and Fhit. It remains to be demonstrated if PLCβ can directly interact with Fhit.

The α2-β4 region of Gα_q_ is essential for the binding of Fhit [15] and other regions may also be required (Fig. 5b). According to the structure of Gα_q_-PLCβ3 complex, PLCβ3 interacts with the α2 and α3 region of Gα_q_ by a helix-turn-helix domain [25] and a similar interaction domain is also present in the Gα_q_/p63RhoGEF complex [27]. Different from PLCβ, p63RhoGEF could not interact with Fhit irrespective of whether activated Gα_q_ was present (Fig. 3b). This tends to suggest that when activated Gα_q_ is bound to an effector other than PLCβ, Fhit is precluded from interacting with Gα_q_. The complete lack of evidence on Fhit and PLCβ competing for activated Gα_q_ further supports the existence of a tripartite complex of Fhit/Gα_q_/PLCβ. A prerequisite for the simultaneous binding of Fhit and PLCβ to activated Gα_q_ is that the two molecules should not use identical regions on Gα_q_ for interaction. This notion is indirectly supported by the results pertaining to the zα2β4QL chimera, which stimulated PLCβ activity as robustly as Gα_16_QL (Fig. 5b) but failed to interact with Fhit beyond that of wild-type zα2β4 (Fig. 5c). It would appear that the conformation of activated zα2β4QL can be recognized by PLCβ but not by Fhit. Although PLCβ has additional contact points (e.g., the α3-β5 region) on Gα_q_ [25], the lack of detrimental effect upon the replacement of the α2-β4 region in the zα2β4QL chimera is rather intriguing and warrant some discussion. In the α2-β4 region of Gα_q_, nine of the ten residues (Q209-K215, H218, C219 and E221) for PLCβ3 interaction are the same with members of Gα_i_ subfamily while the exceptional residue on Gα_i_ subunits corresponding to R214 of Gα_q_ is identical to that of Gα_16_ [25]. Hence, it is possible that substitution of α2-β4 region of Gα_16_ (a Gα_q_ member) with the corresponding region of Gα_z_ (a Gα_i_ subfamily member) would still allow the zα2β4QL chimera to interact productively with PLCβ (Fig. 5b). In contrast, the mere presence of the α2-β4 region of Gα_16_ in the 16α2β4QL chimera was insufficient to support efficient interaction with PLCβ or Fhit. Collectively, these results indicate that the substitution-induced conformational changes on the binding interface of Gα_q_ are tolerated by PLCβ but not by Fhit.

PLCβ is a key molecule in transducing activated G_q_ protein signal to its downstream signal pathways, and similar to G_q_ protein, it also plays complicated roles in regulating cell growth. PLCβ2 expression level is positively correlated with breast cancer [32] and it promotes mitosis and migration of breast tumor cells [33]. On the other hand, in human erythroleukemia cells, PLCβ1 suppresses proliferation probably through regulating cyclin D3 [34]. PLCβ3-deficiency in mice leads to lymphoma and other tumors [35]. The interaction between PLCβs and Fhit as well as the complex formation among Fhit, Gα_q_ and PLCβ reveals new pathway(s) of cell growth inhibition by Gα_q_ and PLCβ.

**Fig. 6:**
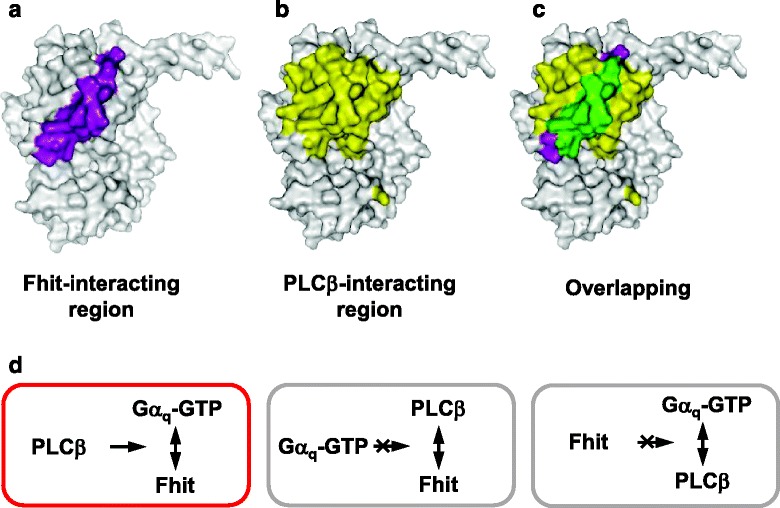
The binding regions of Fhit and PLCβ on activated Gα_q_ surface. Molecular surface of activated Gα_q_ was modeled based on the crystal structures of activated Gα_q_ and PLCβ3 (PDB: 4GNK). **a** The location of the Fhit-interacting α2-β4 regions (Gly208-Asp243, purple) relative to the other domains (white) on Gα_q_ is illustrated. **b** The contact interface of activated Gα_q_ with PLCβ3 is highlighted in yellow. **c** Overlapped binding regions of PLCβ and Fhit on activated Gα_q_ is shown in green. **d** PLCβ increases the interaction between Gα_q_ and Fhit. Gα_q_ does not enhance the interaction between PLCβ and Fhit. And Fhit is unable to strengthen the association of Gα_q_ and PLCβ

Activated Gα_q_ binds to Fhit through the α2-β4 region of Gα_q_ (Fig. 6a and [15]), and it binds to PLCβ through multiple regions of Gα_q_ including the α2 region (Fig. 6b and [25]). The overlapping PLCβ and Fhit binding domain on the activated Gα_q_ is the α2 region (Fig. 6c). As substitution of α2-β4 region of Gα_16_ with the corresponding region of Gα_z_ did not affect the activated Gα_16_-induced PLCβ activation (Fig. 5b), Fhit may bind to this overlapped interacting region on Gα_q_ and change the binding interface between Gα_q_ and PLCβ without affecting the PLCβ activity. The altered binding interfaces of activated Gα_q_ and PLCβ may trigger Gα_q_ and PLCβ to form a ‘clamp’ around Fhit. Beside protein interactions that were found between two protein pairs among Gα_q_, PLCβ and Fhit, PLCβs increased the interaction between Gα_q_ and Fhit. But Fhit or Gα_q_ did not enhance the interaction between PLCβ and Gα_q_ or Fhit, respectively (Fig. 6d). One possibility for increased affinities of Fhit to Gα_q_ by PLCβ is that PLCβ may interact with and stabilize the complex of Fhit and Gα_q_. Another possibility is that when binding to Gα_q_, PLCβ may provide direct binding sites on itself for Fhit which also leads to the formation of a heterotrimeric protein complex. In both possibilities, the activated Gα_q_ recruits PLCβ which acts as a positive regulator for the association of Fhit with activated Gα_q_. In the future, the involvement of PLCβ in the regulation of Fhit by Gα_q_ and their possible roles on cancer therapy should be demonstrated.

## Conclusions

We showed that PLCβ could interact with Fhit, and the expression of PLCβ increased the interaction between Gα_q_ and Fhit. This regulatory effect appears to be unique to PLCβ because other Gα_q_ effectors such as RGS2 and p63RhoGEF could not interact with Fhit. Substitution of the α2-β4 region of Gα_q_ with Gα_i_ did not affect Gα_q_-induced PLCβ activation but eliminated the interaction between Gα_q_ with Fhit. This new Gα_q_/PLCβ/Fhit signaling complex represents a novel pathway of Gα_q_ regulation on tumor suppression.

## Additional file

Additional file 1: Figure S1. Fhit preferentially associates with activated Gα_16_ in HEK293 cells co-expressing PLCβ. HEK293 cells were co-transfected with different combinations of pFlag-CMV2 (V), Flag-Fhit (F), Gα_16_, Gα_16_QL, PLCβ1, PLCβ2 or PLCβ3. After co-immunoprecipitation assay with anti-Flag affinity gel, PLCβ1, 2, 3, Gα_16_, Fhit and α-tubulin were determined by Western blotting. Flag-Fhit pulled down detectably more Gα_16_QL than Gα_16_ (*cf* lanes 1 and 3 of row two), but wild-type Gα_16_ became able to interact with Fhit upon overexpression of PLCβs (*cf* lanes 1 and 2 of row two). All three forms of PLCβ were co-immunoprecipitated with Flag-Fhit but the co-expression of Gα_16_QL did not enhance the interaction between PLCβs and Fhit, rather such interactions were attenuated (Gα_16_ versus Gα_16_QL lanes). (PDF 185 kb)

## Abbreviations

PLCβ: Phospholipase Cβ
IP_3_: Inositol 1,4,5-trisphosphate
Ap_3_A: Diadenosine 5′,5′-P1,P3-triphosphate
RGS: Regulator of G protein signaling
IP: Immunoprecipitation
TCL: Total cell lysate
