# Supplementary figures and images for: Association of activated Gαq to the tumor suppressor Fhit is enhanced by phospholipase Cβ
Source: BMC Cancer. 2015 Oct 24;15:775. doi: 10.1186/s12885-015-1802-z (PMC4619496; doi:10.1186/s12885-015-1802-z)

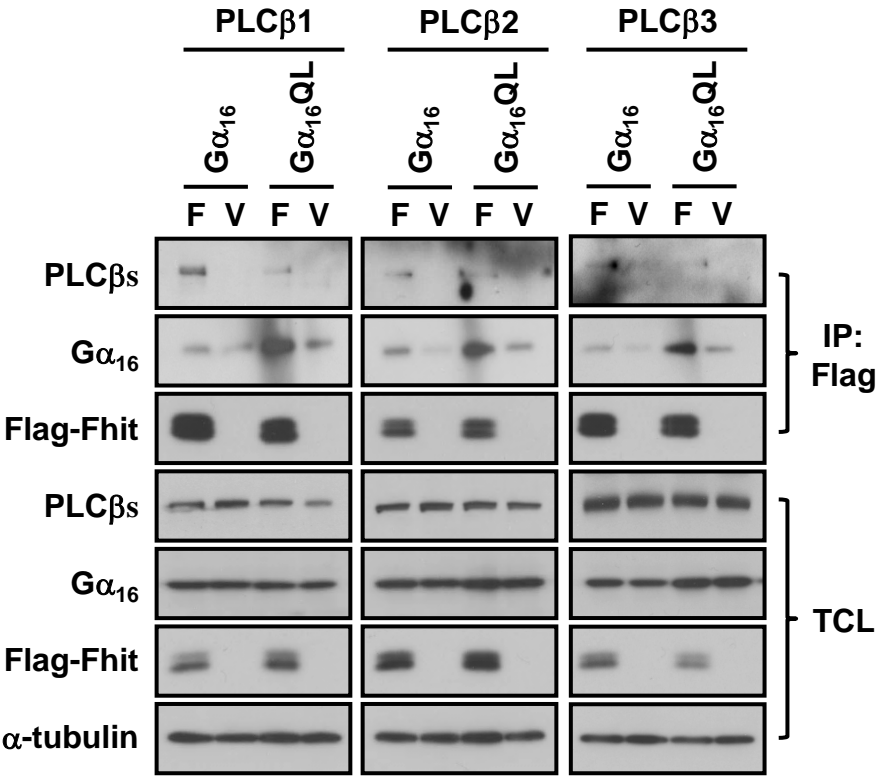

Supplement: Additional file 1: Figure S1. — Fhit preferentially associates with activated Gα16 in HEK293 cells co-expressing PLCβ. HEK293 cells were co-transfected with different combinations of pFlag-CMV2 (V), Flag-Fhit (F), Gα16, Gα16QL, PLCβ1, PLCβ2 or PLCβ3. After co-immunoprecipitation assay with anti-Flag affinity gel, PLCβ1, 2, 3, Gα16, Fhit and α-tubulin were determined by Western blotting. Flag-Fhit pulled down detectably more Gα16QL than Gα16 (cf lanes 1 and 3 of row two), but wild-type Gα16 became able to interact with Fhit upon overexpression of PLCβs (cf lanes 1 and 2 of row two). All three forms of PLCβ were co-immunoprecipitated with Flag-Fhit but the co-expression of Gα16QL did not enhance the interaction between PLCβs and Fhit, rather such interactions were attenuated (Gα16 versus Gα16QL lanes). (PDF 185 kb) [file 12885_2015_1802_MOESM1_ESM.pdf]
